# Palmitate‐Induced Primary Rat Senescent Astrocytes Exhibit Higher Inflammatory Activity and a Distinct Transcriptomic Profile Compared to Reactive Astrocytes

**DOI:** 10.1111/jnc.70164

**Published:** 2025-07-28

**Authors:** Michel López‐Teros, Karla Estephanía Ávila‐Galicia, Raúl Librado‐Osorio, Verónica Jimenez‐Jacinto, Jorge Antonio Garcia‐Álvarez, Georgina Hernández‐Montes, Alejandro Sanchez‐Flores, Adriana Alarcón‐Aguilar, Armando Luna‐López, Mina Königsberg

**Affiliations:** ^1^ Posgrado en Biología Experimental, División de Ciencias Biológicas y de la Salud Universidad Autónoma Metropolitana Unidad‐Iztapalapa Ciudad de México México; ^2^ Laboratorio de Bioenergética y Envejecimiento Celular, División de Ciencias Biológicas y de la Salud Universidad Autónoma Metropolitana Unidad‐Iztapalapa Ciudad de México México; ^3^ Instituto Nacional de Geriatría, San Jerónimo Lídice Ciudad de México México; ^4^ Unidad Universitaria de Secuenciación Masiva y Bioinformática, Instituto de Biotecnología, UNAM Cuernavaca Morelos Mexico; ^5^ Facultad de Ciencias Universidad Nacional Autónoma de México Ciudad de México México; ^6^ Red de Apoyo a la Investigación, Universidad Nacional Autónoma de México (UNAM)‐Instituto Nacional de Ciencias Médicas y Nutrición SZ Ciudad de México México

**Keywords:** astrocytes, gliosis, neuroinflammation, palmitate, senescence

## Abstract

Astrocytes play a crucial role in mediating neuroinflammation, particularly under pathological conditions where they can enter into senescent or gliotic states. This study explored the induction of these two astrocytic states using the same stressor, palmitate, in primary cortical astrocytes. A transcriptomic analysis revealed distinct expression profiles both astrocytes phenotypes. Senescent astrocytes upregulated genes involved in cell cycle arrest and the Senescence‐Associated Secretory Phenotype (SASP), including IGFBP5, CDKN1A, and p53. In contrast, reactive astrocytes upregulated genes related to immune response, complement activation, and inflammation, such as C3, LCN2, and pro‐inflammatory cytokines like IL‐11 and CXCL12. Despite these differences, both astrocytic states shared pro‐inflammatory characteristics, contributing to neuroinflammation. The secretory profiles further distinguished the two states: senescent astrocytes produced higher levels of interleukins, including IL‐6 and IL‐18, indicative of sustained inflammatory responses. Gliotic astrocytes, on the other hand, secreted higher levels of chemokines, such as MCP‐1 and GRO‐α, involved in immune cell recruitment and tissue repair. Senescent astrocytes were more active in cytokine production, while gliotic astrocytes promoted immune responses and tissue repair through chemokine production. Understanding these distinct roles provides valuable insights for developing therapeutic interventions to mitigate neuroinflammation and support healthy brain aging.

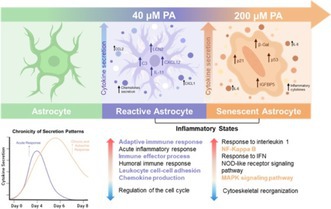

## Graphical abstract

We analyzed the secretome and transcriptome of senescent and reactive cortical astrocytes. Senescent astrocytes secreted more interleukins, while gliotic astrocytes released more chemokines. Astrocytes drive neuroinflammation through both senescence and gliosis.

**Abbreviations**
ABCA14: ATP‐binding cassette sub‐family A member 14
ADCY1: adenylate cyclase type 1
AFAP1L2: actin filament associated protein 1‐like 2
AIPL1: aryl‐hydrocarbon‐interacting protein‐like 1
BTNL10: butyrophilin‐like 10
C6: complement component 6
CAR3: carbonic anhydrase 3
CASQ2: calsequestrin 2
CCL2: chemokine (C‐C motif) ligand 2
CCL3: chemokine (C‐C motif) ligand 3
CCL5: chemokine (C‐C motif) ligand 5
CCL7: chemokine (C‐C motif) ligand 7
CCL20: chemokine (C‐C motif) ligand 20
CD6: cluster of differentiation 6
CD7: cluster of differentiation 7
CDCA3: cell division cycle associated 3
CENPT: centromere protein T
CFB: complement factor B
COL19A1: collagen type XIX Alpha 1 chain
CX3CL1: C‐X3‐C motif chemokine ligand 1
CXCL1: C‐X‐C motif chemokine ligand 1
CXCL11: C‐X‐C motif chemokine ligand 11
CXCL12: C‐X‐C motif chemokine ligand 12
CXCL3: C‐X‐C motif chemokine ligand 3
CXCL6: C‐X‐C motif chemokine ligand 6
DAAs: disease‐associated Astrocytes
EDNRB: endothelin receptor type B
EGR3: early growth response 3
EGR4: early growth response 4
EMP2: epithelial membrane Protein 2
FBXO2: F‐box Protein 2
FPR1: formyl peptide Receptor 1
FXYD2: FXYD domain‐containing ion transport regulator 2
GFAP: glial fibrillary acidic protein
GO: gene ontology
GRO‐α: growth‐regulated oncogene alpha (CXCL1)
HMGA: high mobility group AT‐hook
IFNγ: interferon gamma
IGFBP5: insulin‐like growth factor binding protein 5
IL‐1B: interleukin 1 beta
IL‐4: interleukin 4
IL‐6: interleukin 6
IL‐7: interleukin 7
IL‐10: interleukin 10
IL‐11: interleukin 11
IL‐15: interleukin 15
IL‐17A: interleukin 17A
IL‐18: interleukin 18
IL‐23: interleukin 23
JNK: c‐Jun N‐terminal Kinase
KNSTRN: Kinetochore Localized Astrin/SPAG5 Binding Protein
M‐CSF: macrophage colony‐stimulating factor
MIP‐1A: macrophage inflammatory protein 1 Alpha (CCL3)
MIP‐3A: macrophage inflammatory protein 3 Alpha (CCL20)
MMP2: matrix metallopeptidase 2
MMP13: matrix metallopeptidase 13
MTCO2: mitochondrially encoded cytochrome C oxidase II
MXD3: MAX dimerization protein 3
MYH2: myosin heavy chain 2
NFKB1: nuclear factor kappa B subunit 1
NFKBIA: nuclear factor kappa B inhibitor alpha
NFKBIB: nuclear factor kappa B inhibitor beta
NK cells: natural killer cells
ORA: over representation analysis
PA: palmitic acid
PDMs: peripheral‐derived monocytes
PTGS2: prostaglandin‐endoperoxide synthase 2 (COX‐2)
RANTES: regulated upon activation, normal T cell expressed and secreted (CCL5)
REG3B: regenerating family member 3 Beta
SAHFs: senescence‐associated heterochromatic foci
SASP: senescence‐associated secretory phenotype
SECTM1B: secreted and transmembrane protein 1B
SEPT4: Septin 4
SH2D1A: SH2 domain containing 1A
STAT4: signal transducer and activator of transcription 4
TGF‐B: transforming growth factor Beta
TK1: thymidine kinase 1
TLR2: toll‐like receptor 2
TNNC1: troponin C1
VCAM1: vascular cell adhesion molecule 1
VAT1L: vesicle amine transport 1 Like

## 1 Introduction

Astrocytes represent between 20% and 50% of the Central Nervous System (CNS) cells. They perform important and critical functions, such as neurotransmitter clearance, blood–brain barrier regulation, and stimulating synapse formation, among others (Hasel and Liddelow 2021; Wei and Morrison 2019). Beyond their metabolic and homeostatic roles, astrocytes are key regulators of brain immune responses. When exposed to injury, infection, or other stressful stimuli, astrocytes enter a state called reactive gliosis or astrogliosis (Liddelow et al. 2017). Gliosis is a physiological response of the CNS to injury, infection, or stress. Activated astrocytes increase their proliferation and undergo changes in morphology and physiology to produce a glial scar, isolating the damaged area. They also enhance the production of inflammatory cytokines and chemokines to activate the immune response (Hasel et al. 2021; Escartin et al. 2021). When this response successfully promotes immunosuppression and leads to tissue repair, it is considered to play a beneficial role (Diene et al. 2019). However, if gliosis gets out of control, it can exacerbate inflammatory responses and generate tissue damage (Guillamón‐Vivancos et al. 2015; Clarke et al. 2018; Liddelow et al. 2024). Therefore, astrocytes are crucial modulators of neuroinflammation following acute injuries, such as strokes, and during chronic conditions like Alzheimer's disease and normal aging (Colombo and Farina 2016; Hasel et al. 2021; Giovannoni and Quintana 2020).

Recent transcriptomic studies have revealed the existence of multiple subtypes of astrocytes with different genomic functions, highlighting the functional complexity of these cells (Patani et al. 2023; Giovannoni and Quintana 2020; Escartin et al. 2021). Moreover, in response to various stress stimuli, astrocytes may also enter a state other than gliosis, known as cellular senescence. Senescence is associated with an irreversible cell cycle arrest, in which cells change their morphology and physiology. They become large and flattened, and they modify their gene expression (Hernandez‐Segura et al. 2018; Gorgoulis et al. 2019). The most distinctive feature of senescent cells is the secretion of a complex set of cytokines, chemokines, and other molecules that generate a chronic low‐grade inflammation state associated with the development of diverse pathologies, called Senescent Associated Secretory Phenotype (SASP) (Rodier et al. 2009; Maciel‐Barón, Moreno‐Blas, et al. 2018; Gorgoulis et al. 2019). Senescent astrocytes are known to contribute to neuroinflammation and cognitive decline in murine models (Salas‐Venegas et al. 2023), and their presence has been shown in the brains of patients with neurodegenerative diseases, such as Alzheimer's (AD) and Parkinson's (PD) (Bhat et al. 2012; Chinta et al. 2018).

Neurodegeneration is largely mediated by inflammatory factors, such as interleukin‐1 beta (IL‐1β), IL‐6, tumor necrosis factor‐alpha (TNF‐α), chemokine ligand 2 (CCL2), CCL5, matrix metalloproteinases (MMPs), granulocyte‐macrophage colony‐stimulating factor (GM‐CSF), and glial maturation factor (GMF). These mediators are activated through mitogen‐activated protein kinases (MAPKs) and the nuclear factor kappa B (NF‐kB). Astrocytes release these inflammatory mediators in response to damage, driving neuroinflammation and neurodegeneration (Kempuraj et al. 2016). This cascade of mediators highlights the critical role astrocytes play in modulating the inflammatory environment in neurodegenerative diseases, contributing to both the inflammation and the progressive deterioration of neural tissue. Therefore, in response to various stressful stimuli, astrocytes may become senescent or reactive, and both phenotypes may coexist, promoting the inflammatory responses that are involved in the cognitive decline associated with aging and other neurodegenerative diseases (López‐Teros et al. 2024; Simmnacher et al. 2020; Fróes et al. 2024; Vesga‐Jiménez et al. 2022). Despite this, there are no studies analyzing the interaction between these phenotypes, and this non‐integral approach to the phenomenon may explain the heterogeneity of results observed regarding the effects of senolytics or other drugs on the cognitive decline in aged animal models (Fang et al. 2023, 2024; Rani et al. 2024).

Our group recently published a systematic review evidencing that there are no direct studies comparing the molecules secreted by senescent or activated astrocytes (López‐Teros et al. 2024). Therefore, our aim is to conduct a comparative analysis of the secretory and transcriptomic profiles of senescent and gliotic astrocytes induced to those phenotypes by the same stressor.

Palmitate has been used to induce astrogliosis characterized by increased GFAP and cytokine secretion (Oliveira et al. 2018), generating neuroinflammation and oxidative stress (Fróes et al. 2024; Hou et al. 2022; Sivasubramanian et al. 2024). Palmitate also induces senescence, probably by increasing endoplasmic reticulum stress, DNA damage, and mitochondrial dysfunction (Liang et al. 2024; Ishaq et al. 2022; Sivasubramanian et al. 2024; Schmitt et al. 2024). So, here we established a robust in vitro model using palmitate to independently study senescence and gliosis in primary rat cortex astrocytes.

Our transcriptomic analysis revealed different expression profiles, where senescent astrocytes upregulated genes involved in cell cycle arrest and SASP production, such as IGFBP5, CDKN1A, and p53, while gliotic astrocytes upregulated genes associated with immune response, complement activation, and inflammation, including C3, LCN2, and pro‐inflammatory cytokines like IL‐11 and CXCL12. We also found interesting differences in the secretory profiles. For instance, senescent astrocytes showed greater cytokine production, while gliotic astrocytes secreted more chemokines. Understanding the inflammatory pathways and cytokine production of each phenotype will prove a clearer understanding of how these states contribute to neuroinflammation and their role in neurodegenerative diseases in order to develop insights for therapeutic interventions to mitigate neuroinflammation and to promote healthy brain aging.

## 2 Materials and Methods

### 2.1 Chemicals

All chemicals and reagents were purchased from Sigma Chemical Co. (St. Louis, MO). The reagents obtained from other sources are detailed throughout the text.

### 2.2 Animals

Primary astrocytes were obtained from the cortex of neonatal Wistar rats (RRID:RGD_13508588) (3–7 days old). The animals were provided by the breeding colony of the Universidad Autónoma Metropolitana‐Iztapalapa (UAM‐I); in total, approximately 120 animals were used throughout the study. Animal procedures were always performed following the Mexican Official Ethics Standard 062‐ZOO‐1999 and the National Institutes of Health Guide for the care and use of laboratory animals. This project was approved by the UAM‐I ethics committee (dictate 1706‐2018).

### 2.3 Astrocytes Isolation

Astrocytes were isolated as previously described (Alarcón‐Aguilar et al. 2014; Maciel‐Barón, Morales‐Rosales, et al. 2018; Maciel‐Barón, Moreno‐Blas, et al. 2018). The rats were euthanized by decapitation, according to the NOM‐062‐ZOO‐1999, section 9.5.3.3, and the brain cortexes were transferred to Petri dishes and cut into small fragments. Trypsin (0.01%, 2 mL) (GIBCO cat. no. 25200056) was added and incubated for 5 min at 37°C, 5% CO_2_. To separate the cells, the homogenates were resuspended in MEM‐Medium (MEM/10%, GIBCO cat. no. 61100061; FBS/1%, INVITROGEN cat. no. 16000044‐PRO; Ab‐Am/1.1%, INVITROGEN cat. no. 15240062; Gln/1.5%, SIGMA cat. no. G7513) using a micropipette. The suspension was centrifuged for 15 min at 4°C at 3500 rpm. The pellet was recovered and resuspended in 5 mL of culture medium (MEM/10% FBS/1% Ab‐Am/1.1% Gln/1.5% glucose). The suspension was filtered in a cell strainer (100 μm pore), decanted in a Petri dish, and finally 5 mL of MEM medium was added. Cells were incubated for 24 h at 37°C, 5% CO_2_. The MEM medium was changed every 48 h. Cells were trypsinized and reseeded at a normal density of 4.0 × 10^6^ when the cultures exceeded 80% confluence. Each experiment was performed in triplicate using three independent primary cultures, each prepared by pooling cortices from three different neonatal rat brains.

### 2.4 Palmitate‐BSA Conjugation

Since palmitate is insoluble in aqueous solutions, a conjugation of palmitate with bovine serum albumin (BSA) was done to create a water‐soluble reagent that could be absorbed and utilized by the cells. Palmitic acid 5 mM (SIGMA cat. no. P.0500) was dissolved in 1% DMSO, and BSA 0.85 mM (SIGMA cat. no. A7906) was dissolved in phosphate‐buffered saline (PBS). Both solutions were mixed in a water bath at 65°C with continuous agitation. This new solution formed of palmitic acid‐BSA (molar ratio 6:1) was added to the cells and will further be called palmitate (PA) (Posadas‐Rodríguez et al. 2020). PA was freshly prepared on the day of the astrocyte's treatments to induce senescence and gliosis.

### 2.5 PA Internalization and Cytotoxic Effect

Prior to the standardization of the senescence and gliosis induction model, we verified that the PA actually entered the cells and ruled out the concentrations at which it was cytotoxic. Therefore, astrocytes were treated for 24 h with different PA concentrations (20, 40, 60, 80, 100, 200, 300, and 400 μM). Red Oil staining and cell survival were evaluated. Astrocytes were washed with PBS and fixed in 4% paraformaldehyde for 15 min. After two more washes, cells were permeabilized with propanol v/v 60% and stained with freshly prepared 0.2% Oil Red O solution (ORO) for 20 min. Subsequently, astrocytes were stained with hematoxylin for 5 min. The images were obtained under a ZEISS AXIO Observer Z1 Microscope (Posadas‐Rodríguez et al. 2020). A trypan blue assay (López‐Diazguerrero et al. 2006) after 24 h was performed to determine astrocyte survival after PA treatment.

### 2.6 Astrocyte's Senescence and Gliosis Induction

Astrocytes were seeded at 1–2 × 10^5^ densities in 6‐well plates (Corning, ActonMA, USA), and a new PA dose–response curve was performed, this time to determine the optimal concentration to induce cellular senescence or gliosis (20–400 μM PA for 24 h). Cell growth percentage was evaluated on days 2, 4, and 6 by counting the number of cells each day using the trypan blue assay (López‐Diazguerrero et al. 2006). The goal was to determine the concentration that would stop proliferation for senescence or increase it for gliosis. Astrocytes were used in early passages (5–7) to avoid replicative senescence. Cellular proliferation and SA‐β‐Gal activity were determined in all PA‐treated cells.

### 2.7 Senescence‐Associated B‐Galactosidase (SA‐B‐Gal) Activity and Proliferation Rate

PA‐treated astrocytes were fixed with 4% paraformaldehyde at days 2, 4, and 6 after treatment. SA‐β‐Gal activity was determined as described by Dimri et al. (1995). X‐gal (PROMEGA cat. no. V3941) treated astrocytes were subsequently stained with Safranin to easily differentiate the astrocytes. One hundred astrocytes were counted to report the percentage of SA‐β‐Gal‐positive cells.

On days 2, 4, and 6 after PA treatment, cells were detached with trypsin, stained with trypan blue, and counted. The population percentage and proliferation rate were determined as described by Maciel‐Barón et al. (2016).

### 2.8 Senescence and Gliosis Markers Evaluation

Astrocytes were seeded on coverslips at a density of 2 × 10^5^ in 6‐well plates. Senescence and gliosis inductions were performed at 24 or 48 h after cell seeding. Six days after the induction, astrocytes were fixed with 4% paraformaldehyde for 15 min and washed twice with 0.05% PBS‐Tween. Astrocytes were permeabilized with 0.01% PBS‐Triton for 30 min and subsequently incubated with 2 mL of protein blocker for 30 min and washed twice again with 0.05% PBS‐Tween. PA‐treated astrocytes were incubated for 1 h with specific antibodies used as markers for senescence and gliosis, which are described below. After that, three washes were performed with 0.05% PBS‐Tween, and cells were incubated for 45 min with the respective secondary antibody (Alexa Fluor 594 AffiniPure Donkey Anti‐Mouse IgG (H + L) ABCLONAL cat. no. AS035 & AS054). Cells were washed again three times with PBS‐Tween, and 10× Phalloidin was added for 20 min. Finally, astrocytes were washed three times and mounted with DAPI (4′,6‐diamidino‐2‐phenylindole, dihydrochloride) (cat. no. 62247; Thermo Scientific) on glass slides. The images were obtained from a confocal microscope LSM‐META‐Zeiss Axioplan 2 (20×). The relative intensities of the signals were measured using ImageJ software.

The antibodies used to corroborate cellular senescence were γH2A.X, GLB, and Lamin B1, all of them at 1:100 dilution (sc‐517348, sc‐65670, and sc‐374015, cat. no.; Santa Cruz Biotechnology, respectively). These markers were selected based on their established association with key features of the senescent phenotype: GLB (β‐galactosidase) is a widely used indicator of lysosomal activity in senescent cells, while γH2A.X reflects persistent DNA damage response (Burton and Krizhanovsky 2014). Lamin B1 was included due to its consistent downregulation during senescence, as reported by Freund et al. (2012).

For reactive gliosis, the antibodies used were C3, GFAP, and S100A10 (all at 1:100 dilution; sc‐33673, and sc‐81153; Santa Cruz Biotechnology; sc‐28294, respectively). These antibodies were selected based on the work by Liddelow et al. (2017), who identified them as reliable markers of reactive astrocyte subtypes in response to neuroinflammation and injury.

### 2.9 RNA‐Seq Analysis

For the RNA‐seq analysis, 3 biological replicates for senescence and gliosis conditions were used. RNA extraction for each replicate was performed using the Zymobiomics RNA Miniprep Kit. Library construction was performed by following the Illumina TruSeq stranded mRNA protocol. Sequencing was carried out on an Illumina NextSeq 500 platform using the NextSeq 500/550 High Output Kit v2.5 (150 cycles) for 2 × 75 paired‐end sequencing. Yields exceeded 10 million sequences per replicate, with an average PHRED quality score above Q32 across all sequences. No adapter contamination was detected, so no sequences were discarded based on quality or adapter presence. Quality and adapter detection were performed using FastQC v0.12.0 (http://www.bioinformatics.babraham.ac.uk/projects/fastqc).

Bioinformatic analysis began with a paired alignment of all replicates and conditions against the rat genome downloaded from NCBI (GCF_000001895.5_Rnor_6.0_genomic.fna.gz). The genome was indexed using a k‐mer size of 20 and a step size of 13 for the short‐read alignment program smalt‐0.7.6, with an alignment similarity threshold of 80%. For each replicate a BAM file was obtained and optical duplicates were marked using MarkDuplicates VN:2.18.14‐SNAPSHOT. Coverage was calculated with coverageBed from the bedtools2‐2.27.1 package and a count table with all replicates was generated using an in‐house Perl script. Four methods were used for differential expression analysis: NOISeq, DESeq2, EdgeR, and limma. For each method a cutoff value <= 0.05 of adj‐pval or FDR (False Discovery Rate) was considered for parametric methods and for the non‐parametric method, NOISeq the equivalent metric Prob > = 0.9 was used to determine a differentially expressed gene (DEG). Then, DEGs for each method were compared, and we reported the intersection for the 4 method DEG lists, which were generated based on log_2_ FC values calculated from RPKM, comparing the treatment conditions to the untreated control condition.

Hierarchical clustering and heatmaps representing z‐scores were created using the seaborn package in Python. The senescence marker gene list was composed from the “senescence” term from the Rat Genome Database (RGD), CellAge (the cellular senescence gene database, RRID:SCR_007700), and the senescence marker genes mentioned in Simmnacher et al. (2020) and Moiseeva et al. (2023). The gliosis marker gene list was derived from Matusova et al. (2023), Simmnacher et al. (2020), Soung and Klein (2020), and Liddelow et al. (2017). To identify inflammatory markers, the study drew on the findings reported by Simmnacher et al. (2020), complemented by a search on the AmiGO2 platform. The search was filtered specifically for *Rattus norvegicus* and included the following keywords: “cytokines,” “inflammatory response,” “cytokine production,” “regulation of cytokine production,” “positive regulation of neuroinflammation,” “cytokine‐mediated signaling pathway,” and “cytokine activity.” For immune‐related markers, the search strategies focused on terms such as “immune response,” “activation of immune response,” and “adaptive immune response.” Regarding metabolic markers, the selection was based on those previously described in the literature, referencing studies such as Cali et al. (2024), Xiao and Hu (2014), Kagawa et al. (2020), Bellaver et al. (2017), and Matias et al. (2023).

GSEA was performed using GSEA v3.0 software (RRID:SCR_003199). GSEAPreranked analysis was conducted on the ranked list of log_2_ FC values of all expressed genes using the Hallmarks gene sets (v6.2) from the MSigDB database (RRID:SCR_016863).

Since we performed a differential expression analysis using four different methods (EdgeR, DESeq2, limma and NOIseq) a cutoff value <= 0.05 for the adjusted *p*‐value or equivalent (like FDR) was used to consider a gene as differentially expressed (DEG). In the case of the non‐parametric method NOISeq the equivalent Prob value was used, and the cutoff value was > = 0.9. Then, DEGs for each method were compared and we reported the intersection for the four methods with the assumption that four methods can't reproduce the same mistake reporting a gene as differentially expressed.

For better visualization and data collection, we used the web platform Integrative Differential Expression Analysis for Multiple Experiments (IDEAMEX) (Jiménez‐Jacinto et al. 2019). The gene list with all the values used for the calculations, including the adj‐pval or equivalent are included in the following link: (http://www.uusmb.unam.mx/ideamex/Project_MKonigsberg_2023_03_07_17_25_46.Reduce3/index.php). IDEAMEX allows integrated analysis of RNA‐Seq data. On the main page, graphs such as count per million (CPM), boxplots, density, multidimensional scaling (MDS), principal component analysis (PCA), among others, are available. The results generated by each of the analytical platforms used: edgeR, DESeq2, Limma and NOISeq can be consulted in the menu on the left side. For the final analysis, the results obtained with DESeq2 were selected. Clicking on the name of the analysis, the corresponding databases are displayed, which include the *p*‐value values for each gene. Treatments were named with the following acronyms: CTRL, Control; PAIPS, Palmitate Astrocyte Induced Premature Senescence; PAIG, Palmitate Astrocyte Induced Gliosis.

### 2.10 Secretory Profile Analysis

A total of 23 cytokines were quantified using the Bio‐Plex Pro Rat Cytokine 23‐Plex Assay (Bio‐Plex Cat. no. 12005641), using the methodology described by Maciel‐Barón, Morales‐Rosales, et al. (2018); Maciel‐Barón, Moreno‐Blas, et al. (2018). The conditioned media (CM) were evaluated on days 4, 6, and 8. Forty‐eight hours before collecting the conditioned media, cells were incubated with MCDB105 medium supplemented with 1% Ab‐Am. After 48 h, the CM was collected and centrifuged at 3500 rpm to remove cellular debris and was concentrated 10‐fold using Millipore Amicon Ultra centrifugal filter units, following the manufacturer's instructions, and were stored at −80°C until use. The Bio‐Plex assay was performed according to the manufacturer's instructions. Data was acquired on a Luminex instrument, and analyte concentrations were measured according to the manufacturer's recommendations. The secretory profile was performed in triplicate from three independent cultures, and each data point was normalized against the cell number.

### 2.11 Statistical Analysis

Data are presented as mean ± standard deviation (SD) from at least three independent experiments in at least three technical triplicates, using primary astrocyte cultures derived from different animals (biological replicates), each prepared by pooling cortices from three different neonatal rat brains (no sample size calculation was performed). Data normality was assessed using the Shapiro–Wilk test in GraphPad Prism version 8.0.1 prior to applying parametric tests. No formal outlier test was performed, and all data points were included in the statistical analysis in order to preserve the biological variability inherent to primary astrocyte cultures. Statistical comparisons were performed using one‐way or two‐way ANOVA, as appropriate, followed by a Tukey–Kramer post hoc test. A *p*‐value < 0.05 was considered statistically significant. The definition of *n* is provided in each figure legend. Additionally, a statistical report including all the individual data from each analysis is provided in the Appendix S1.

## 3 Results

### 3.1 Different Concentrations of Palmitate Induce Premature Senescence or Gliosis in Astrocytes

**FIGURE 1:**
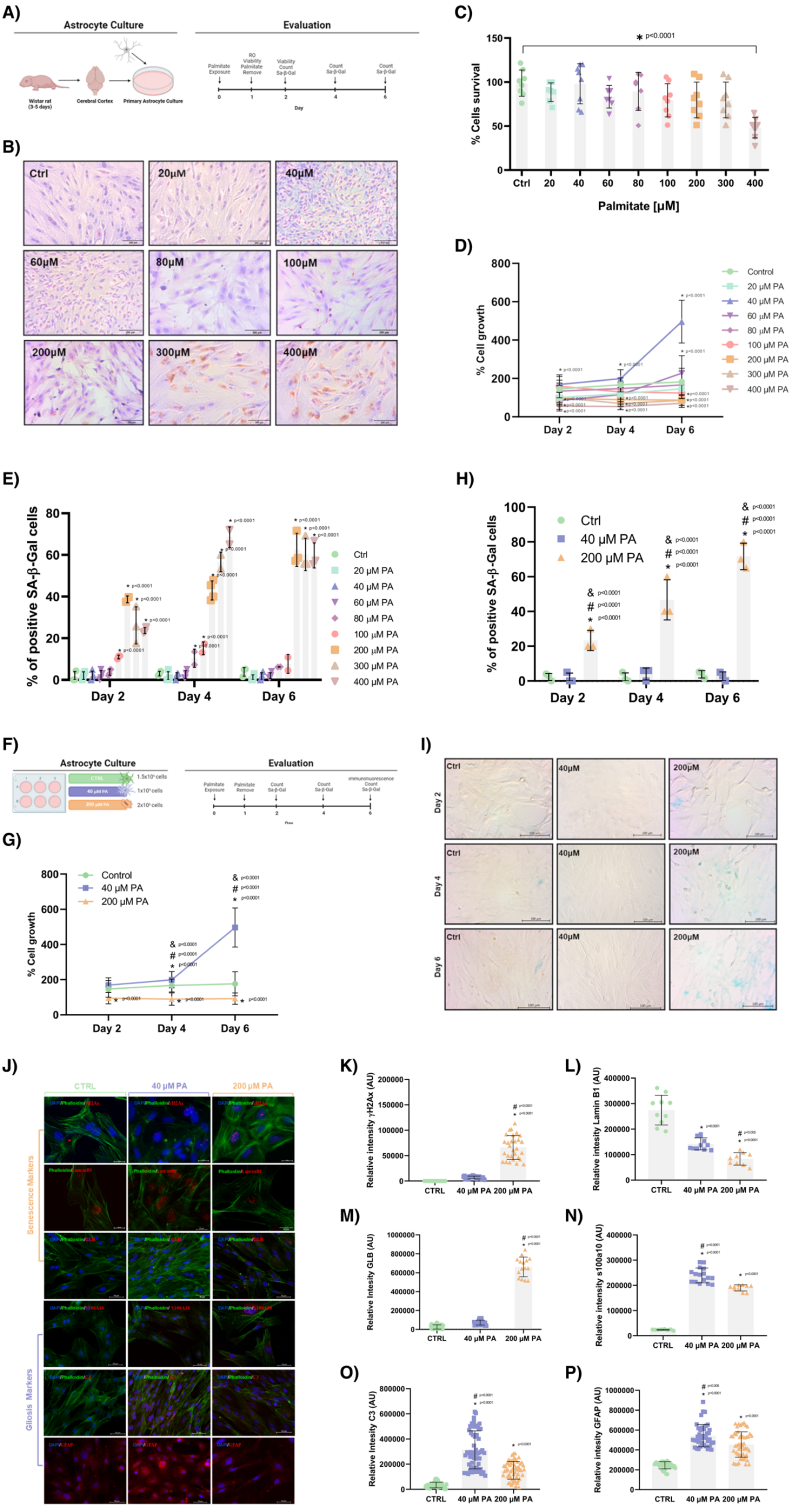
Palmitate induces either gliosis or premature senescence in primary rat astrocytes depending on concentration. (A) An in vitro model was designed to induce reactive gliosis and cellular senescence by exposing primary rat astrocytes to different palmitate (PA) concentrations. Experimental evaluations were performed on days 2, 4, and 6 post‐treatment. (B) The initial step was to confirm intracellular uptake of PA using Oil Red O staining at 24 h, revealing dose‐dependent lipid accumulation. (C) Cell viability assays showed that concentrations ≥ 300 μM significantly compromised cell survival, and were therefore excluded from further analysis. (ANOVA: *F* = 5.63, *p* < 0.0001, Tukey's: *Q* = 8.06 df = 60, *p* < 0.0001). (D) Proliferation was evaluated as a hallmark of both senescence and gliosis. Concentrations of 200, 300, and 400 μM led to reduced proliferation, while 40 μM significantly increased proliferation from day 2 and on. (ANOVA: *F* = 21, *p* < 0.0001, Tukey's: *Q* = 8.07 df = 351, *p* < 0.0001). (E) SA‐β‐Gal activity assays revealed that 200, 300, and 400 μM PA induced > 60% SA‐βgal‐positive cells by day 4. The 40 μM PA concentration did not produce SA‐βgal‐positive cells. (ANOVA: *F* = 21.54, *p* < 0.0001, Tukey's: *Q* = 27.26 df = 58, *p* < 0.0001). (F) Comparison of cellular proliferation or arrest in response to 200 or 40 μM PA. (G) Initial seeding densities were adjusted according to the expected proliferative behavior of each condition to allow consistent comparisons across timepoints (ANOVA: *F* = 45.09, *p* < 0.0001, Tukey's: *Q* = 27.47 df = 116, *p* < 0.0001). (H–I) Proliferation and SA‐βgal assays using 40 and 200 μM PA confirmed the distinct phenotypic profiles observed in the concentration curve (ANOVA: *F* = 18.74, *p* < 0.0001, Tukey's: *Q* = 22.24 df = 18, *p* < 0.0001). (J) Representative images of SA‐βgal staining illustrate the morphological and population changes over time for each condition. (K–P) Phenotypic validation was performed by immunofluorescence using specific markers. Astrocytes treated with 200 μM PA showed increased SA‐βgal and γH2AX staining, and decreased Lamin B1, confirming the senescent phenotype. Astrocytes exposed to 40 μM PA showed increased expression of C3, GFAP, and S100A10, validating the reactive gliosis phenotype. (K–M) Quantification of relative fluorescence intensity for Lamin B1 (ANOVA: *F* = 63, *p* < 0.0001, Tukey's: *Q* = 15.10 df = 27, *p* < 0.0001), γH2AX, (ANOVA: *F* = 124.1, *p* < 0.0001, Tukey's: *Q* = 19.71 df = 63, *p* < 0.0001), and GLB (ANOVA: *F* = 124.1, *p* < 0.0001, Tukey's: *Q* = 65.29 df = 90, *p* < 0.0001) confirmed the senescent phenotype induced by 200 μM PA. (N–Q) Quantification of S100A10 (ANOVA: *F* = 274.3, *p* < 0.0001, Tukey's: *Q* = 32.89 df = 31, *p* < 0.0001), C3 (ANOVA: *F* = 100.4, *p* < 0.0001, Tukey's: *Q* = 19.72 df = 158, *p* < 0.0001), and GFAP (ANOVA: *F* = 79.87, *p* < 0.0001, Tukey's: *Q* = 17.47 df = 126, *p* < 0.0001) confirmed the gliotic phenotype induced by 40 μM PA. Immunofluorescence intensity data were normalized to cell number on day 6 to ensure accurate comparisons between conditions at this key timepoint. Data are presented as mean ± standard deviation (SD) from *n* = 3 independent biological replicates (each derived from a pool of three neonatal rat cortices), with three technical replicates per culture. Significance: *p* < 0.0001 compared to control (*), gliotic astrocytes (#), or the previous day of the same treatment (&).

Figure 1A describes the experimental design used to validate the in vitro model. PA entry into the cells was confirmed by the Red Oil assay (Figure 1B), along with the cellular survival at 24 h, to rule out the concentrations at which PA was cytotoxic (Figure 1C). The concentrations above 400 μM significantly reduced cell survival compared to the control, while 100 and 200 μM concentrations did not show significant differences. Therefore, the concentrations between 20 and 400 μM were tested for senescence or gliosis induction. Then, astrocytes proliferation was evaluated on days 2, 4, and 6 following PA exposure in order to find the concentration that would stop proliferation for senescence induction and the one that would increase it for gliosis induction (Figure 1D). The three higher concentrations (200, 300, and 400 μM) stopped cellular proliferation after 6 days; however, 300 and 400 μM also significantly reduced cell numbers compared to the control from day 2 onwards. None of the concentrations below 100 μM PA had cytotoxic effects. In contrast, the 40 μM PA concentration significantly increased cell proliferation compared to control from day 2 of PA exposure, so this concentration was chosen as a prospective for gliosis induction (Figure 1D).

The SA‐β‐Gal assay was used at this point to evaluate the senescent state. Figure 1E shows that 200, 300, and 400 μM PA induced 50%–70% of SA‐β‐Gal positive cells since day 4 and on. So, these three concentrations were identified as potential inducers of senescence, but considering that 300 and 400 μM PA significantly decreased the cell population, the 200 μM concentration was chosen as the optimal concentration to induce senescence (Figure 1D,E). Up to this point, 200 and 40 μM PA were chosen as potential inducers of senescence and gliosis respectively (Figures G, H, and I); so, to confirm the senescent or gliotic states, senescence and gliosis markers were evaluated (Figure 1F,J). Approximately 80% of the astrocytes exposed to 200 μM PA exhibited an increase in γH2AX (Figure 1K) and GLB (β‐Gal) (Figure 1M), along with a decrease in Lamina B1 (Figure 1L). While astrocytes treated with 40 μM PA showed increased S100A10 (Figure 1N), C3 (Figure 1O), and GFAP (Figure 1P). Astrocytes exposed to 200 μM PA showed no gliosis markers and those exposed to 40 μM PA showed no senescence markers (Figure 1J). Therefore, 200 μM PA was selected as the optimal concentration for inducing senescence in astrocytes, while 40 μM PA was chosen to induce gliosis.

### 3.2 Transcriptomic Differences Between Senescent and Reactive Astrocytes

**FIGURE 2:**
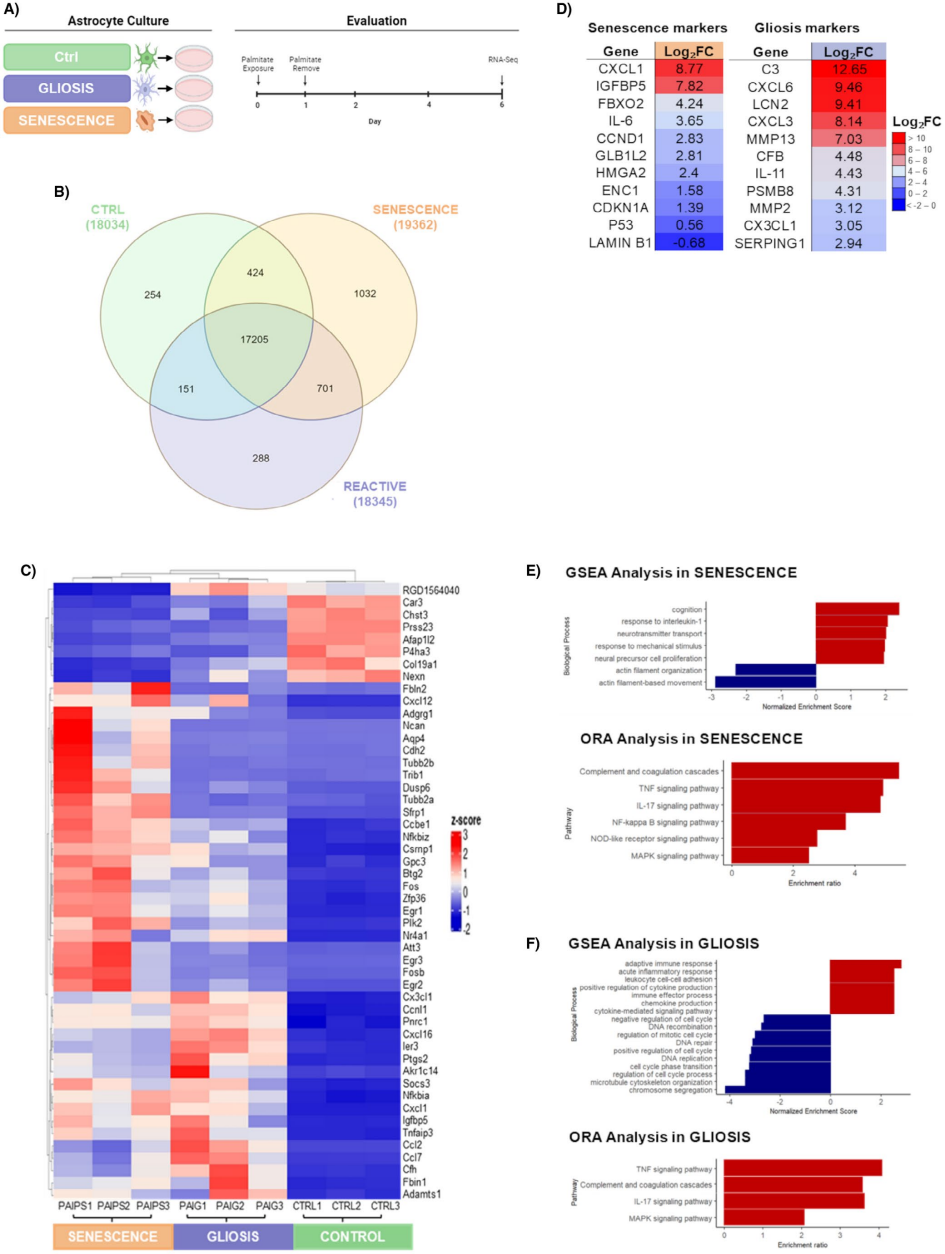
Transcriptomic profiling reveals distinct gene expression signatures in gliotic and senescent astrocytes. RNA sequencing was performed to characterize and compare the transcriptional profiles of astrocytes treated with either 40 or 200 μM PA, previously established to induce reactive gliosis or cellular senescence, respectively. (A) Experimental model: RNA was extracted at day 6 post‐treatment with 40 or 200 μM PA, based on prior phenotypic validation. (B) Venn diagram depicting differentially expressed genes (DEGs) that are unique to or shared between gliotic, senescent, and control astrocytes. Senescent astrocytes exhibited 1032 exclusive DEGs, gliotic astrocytes 288, while 701 genes were shared between both conditions. (C) Heatmap showing the top 50 upregulated genes in senescent astrocytes relative to gliotic and control groups. The clustering analysis revealed the formation of three transcriptionally distinct phenotypes. Notably, senescent and gliotic astrocytes were more similar to each other than to control astrocytes. (D) Based on the observed segregation into three distinct groups, we selected and examined the expression of canonical markers for both phenotypes as described in the literature. Senescence‐associated genes (Cdkn1a, Glb1, Trp53) and gliosis‐related genes (C3, Gfap, S100a10) showed differential expression across groups, supporting the establishment of distinct reactive and senescent profiles. (E) Gene Set Enrichment Analysis (GSEA) and Over‐Representation Analysis (ORA) of senescent astrocytes revealed significant enrichment in pathways related to DNA damage response, inflammation, and cell cycle arrest. (F) GSEA and ORA of gliotic astrocytes showed enrichment in immune‐related pathways, extracellular matrix remodeling, and reactive processes. DEGs were identified using four independent methods (DESeq2, edgeR, limma, and NOISeq), and only overlapping genes were considered. All samples were aligned to the rat genome, and log_2_ fold changes were computed from normalized RPKM values. Data represent *n* = 3 independent cultures (each from pooled cortices of 3 neonatal rats), with 3 technical replicates each. Statistical significance: *p* < 0.05 compared to control (*), gliotic astrocytes (#), or across time (&); FDR‐adjusted*.

Six days after senescence or gliosis induction, a transcriptomic comparison was conducted (Figure 2A). Differentially expressed genes (DEGs) were identified for each group: 288 in reactive astrocytes, 1032 in senescent astrocytes, and 254 in CTRL (Figure 2B). A total of 701 genes were shared between reactive and senescent astrocytes, indicating similarities between these two groups and distinguishing them from the CTRL group.

The heatmap in Figure 2C illustrates the top 50 DEGs identified for reactive, senescent astrocytes, and CTRL, showing the differences based on hierarchical clustering. Senescent astrocytes clustered closer to reactive astrocytes than to CTRL, indicating a smaller subset of genes showing differential expression between these groups compared to CTRL.

The heatmap highlights the top 50 DEGs identified for both senescence and gliosis compared to CTRL (Figure 2C). An increased expression is shown in red, while a decreased expression is shown in blue relative to CTRL. DEGs in senescent astrocytes did not show the same expression patterns as reactive astrocytes or CTRL, demonstrating the different expression profiles of each group.

### 3.3 Senescent and Gliotic Astrocytes Upregulate Specific Genes and Pathways

The transcriptomes of reactive and senescent astrocyte models indicate the presence of senescence and gliosis markers (Figure 2D). Senescence markers in senescent astrocytes included regulators of the cell cycle and its arrest (FBXO2, IGFBP5, ENC1, CCND1), pathways related to p53 (p53) pathways related to p21 (CDKN1A), chromosomal mobility (HMGA2), genes associated with the SASP (IL‐6, CXCL1, IL‐1α), nuclear membrane loss (Lamin B1), and β‐Galactosidase‐related genes (GLB1L2). Reactive astrocytes showed inflammatory markers (GBP2, PSMB8, SRGN, AMIGO2, LCN2), complement activation (C3, C6, CFB, MX1, SERPING1), and pro‐inflammatory cytokine production (IL‐11, CXCL3, CXCL12, CXCL6, CX3CL1, MMP2, MMP13). The protein expression of some of these genes, such as Lamin B1, GLB1, and C3, is consistent with the findings described in Figure 2.

### 3.4 Key Genes and Pathways in Senescent and Gliotic Astrocytes

Using the Gene Set Enrichment Analysis (GSEA) and the Over‐Representation Analysis (ORA), we identified that senescent astrocytes are involved in pathways related to cognition, interleukin response, neurotransmitter transport, response to mechanical stimuli, complement cascades, and signaling pathways, such as TNF, IL‐17, NF‐κB, MAPK, and NOD‐like receptor, all of which were upregulated. Conversely, processes associated with cytoskeletal organization were found to be downregulated (Figure 2E). In contrast, reactive astrocytes showed enrichment in processes, such as adaptive immune response, acute inflammatory response, leukocyte cell–cell adhesion, and the production of cytokines and chemokines, all of which were upregulated. However, processes related to cell cycle regulation and DNA repair were downregulated, highlighting key functional differences between the two cellular states (Figure 2F).

**FIGURE 3:**
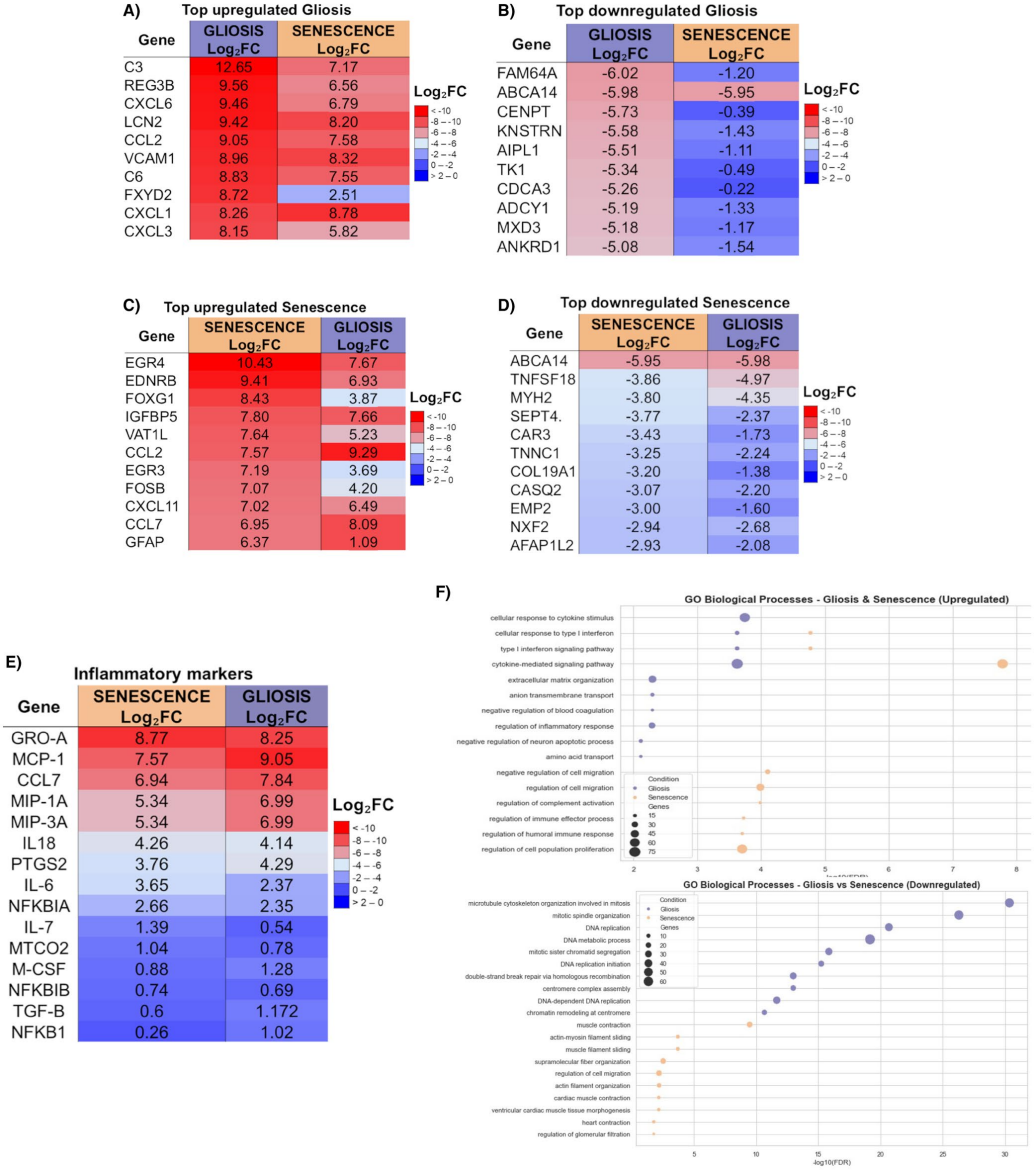
Top differentially expressed genes and inflammation‐related signatures in senescent and gliotic astrocytes. RNA‐Seq analysis was used to identify the most differentially expressed genes between gliotic and senescent astrocytes on day 6 post‐treatment. Top 10 most upregulated (A) and downregulated (B) genes in gliotic astrocytes, with their relative expression levels in the senescence condition for comparison. Top 10 most upregulated (C) and downregulated (D) genes in senescent astrocytes, compared to their expression in gliotic astrocytes. (E) Expression levels of selected inflammation‐related genes revealed distinct cytokine and chemokine profiles between the two phenotypes. (F) Gene Ontology (GO) enrichment analysis of biological processes associated with the upregulated and downregulated genes in both conditions revealed distinct functional programs: Gliotic astrocytes were enriched in immune response, chemotaxis, and ECM remodeling pathways, while senescent astrocytes showed enrichment in cell cycle arrest, DNA damage response, and chromatin organization. Data represent *n* = 3 independent biological replicates, each consisting of primary astrocyte cultures derived from pooled cortices of three neonatal rats, with three technical replicates per culture. Statistical significance was determined by intersecting DEG results from four independent methods (DESeq2, edgeR, limma, and NOISeq), using adjusted *p*‐value or probability thresholds as detailed in Methods. Significance: *p* < 0.05 compared to control (*), gliotic astrocytes (#), or across time (&); FDR‐adjusted*.

The top 10 up‐ and downregulated DEGs in both phenotypes were also analyzed and compared (Figure 3). The top upregulated DEGs in reactive astrocytes included complement activators (C3, C6), inflammation markers (REG3B, LCN2), cytokine production (CCL2, CXCL1, CXCL6, CXCL3), cell adhesion (VCAM1), and ion regulation (FXYD2) (Figure 3A). Additionally, downregulated genes were cell cycle regulators (FAMG4A, CENPT, KNSTRN, AIPL1, TK1, CDCA3, MXD3), cell signaling genes (ADCY1), and lipid transport genes (ABCA14) (Figure 3B).

The top 10 upregulated DEGs in senescent astrocytes comprised cell cycle arrest markers (FOSB, IGFBP5), inflammatory genes (CCL7, LCN2, CCL2, EDNRB, CXCL11, C6), ion regulation genes (VAT1L), and synapse regulation genes (EGR4, EGR3) (Figure 3C). The top 10 downregulated genes included those involved in cell signaling (EMP2), cytoskeleton organization (MYH2, SEPT4, CAR3, TNNC1, COL19A1, AFAP1L2), lipid transport (ABCA14), and calcium levels (CASQ2) (Figure 3D).

### 3.5 Key Inflammatory Genes and Pathways in Senescent and Gliotic Astrocytes

An analysis of the previously described inflammatory markers was also performed to compare both phenotypes (Figure 3E). Genes highly differentially expressed in senescent astrocytes included MTCO2, NFKBIA, NFKBIB, GRO‐α, IL‐7, IL‐18, and IL‐6, while genes such as CCL7, TGF‐B, NFKB1, PTGS2, M‐CSF, MCP‐1, MIP‐1A, and MIP‐3A were highly expressed in reactive astrocytes.

The Gene Ontology (GO) analysis (Figure 3F) revealed that both models shared involvement in biological processes such as cellular response to cytokine stimulus, cellular response to type I interferon, and cytokine‐mediated signaling pathway, all of which were upregulated with moderate statistical significance in gliosis (−log_10_(FDR) between 3 and 5), and high in senescence (−log_10_(FDR) > 7). Senescent astrocytes showed an increased number of genes associated with processes such as cell proliferation and complement activation, whereas reactive astrocytes showed moderate activation of inflammatory processes. In down‐regulated processes, senescent astrocytes showed changes in key cell cycle functions such as DNA replication and double‐strand break repair, with −log_10_(FDR) values reaching up to 30 and a high representation of genes. In gliosis, changes were observed mainly in structural processes such as muscle contraction and supramolecular fiber organization, with a lower statistical significance (−log_10_(FDR) between 5 and 10). Although both conditions shared processes related to inflammation and structural organization, senescence was more involved in inflammatory processes such as regulation of the immune response and complement activation, showing a higher statistical significance and a greater number of associated genes. In contrast, gliosis mainly affected structural processes and inflammatory mechanisms of lower intensity. In addition, senescence was characterized by a profound alteration of cell cycle functions, whereas gliosis had a more localized impact on muscle functions and fiber organization.

### 3.6 Cytokine Secretion in Senescent and Gliotic Astrocytes

**FIGURE 4:**
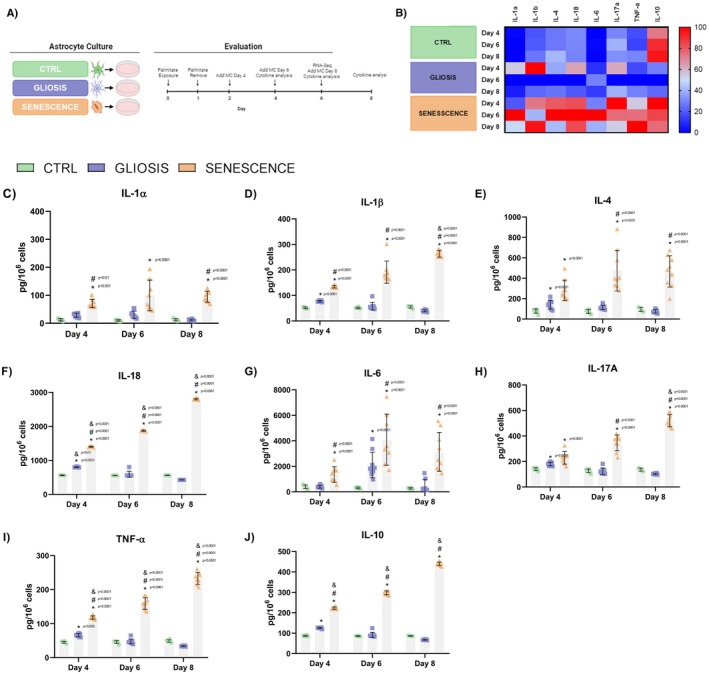
Secretory cytokine profile of reactive and senescent astrocytes on days 4, 6, and 8. The temporal cytokine secretion pattern of astrocytes undergoing gliosis or senescence was evaluated using a multiplex immunoassay. (A) Experimental model: Conditioned media were collected on days 2, 4, and 6 post‐induction and incubated for 48 h before analysis using the Bio‐Plex Pro Rat Cytokine 23‐plex Assay. (B) Heatmap summarizing differential cytokine secretion (IL‐1α, IL‐1β, IL‐4, IL‐18, IL‐8, IL‐17a, TNF‐α, IL‐10) across conditions and timepoints. (C–J) Quantification of individual cytokines revealed dynamic and condition‐specific secretion profiles. Notably, senescent astrocytes showed sustained elevation of IL‐1α, IL‐6, and IL‐10, while gliotic astrocytes secreted higher levels of IL‐1β, IL‐4, and IL‐17a, particularly at earlier timepoints. (ANOVA: *F* = 60.29, *p* < 0.0001, Tukey's: *Q* = 32.89 df = 58, *p* < 0.0001). Data represent *n* = 3 independent biological replicates, each consisting of primary astrocyte cultures derived from pooled cortices of three neonatal rats, with three technical replicates per culture. Concentration values were normalized to cell number to account for differences in cell density across conditions.

Given the upregulation of genes involved in cytokine and chemokine production, a direct quantification of them was conducted on days 4, 6, and 8 for each group (Figure 4A). Cytokine secretion per pg of mL was analyzed. The quantification of each cytokine and chemokine is provided in Table S1. As shown, the concentration of cytokines and chemokines was higher in reactive astrocytes; however, when normalized by cell number, the results were markedly different, as shown in Figure 4.

A heatmap was generated to illustrate the cytokine secretion profile, which changed over time (Figure 4B). In senescent astrocytes, cytokine secretion generally peaked on day 6, particularly for IL‐1α (Figure 4C) and IL‐6 (Figure 4G), while some cytokines, such as IL‐17α (Figure 4H) and IL‐10 (Figure 4J), decreased over time. Reactive astrocytes showed an increase in cytokine secretion on day 4, which declined by day 6 and 8. Significant differences in cytokine secretion were observed between senescent and reactive astrocytes on day 4 for IL‐1α, IL‐4, and IL‐17α, with senescent astrocytes showing higher secretion. By day 6, senescent astrocytes showed an increased secretion of IL‐1α, IL‐4, IL‐18, IL‐6, IL‐17α, TNF‐α, and IL‐10 (Figure 4C–J).

**FIGURE 5:**
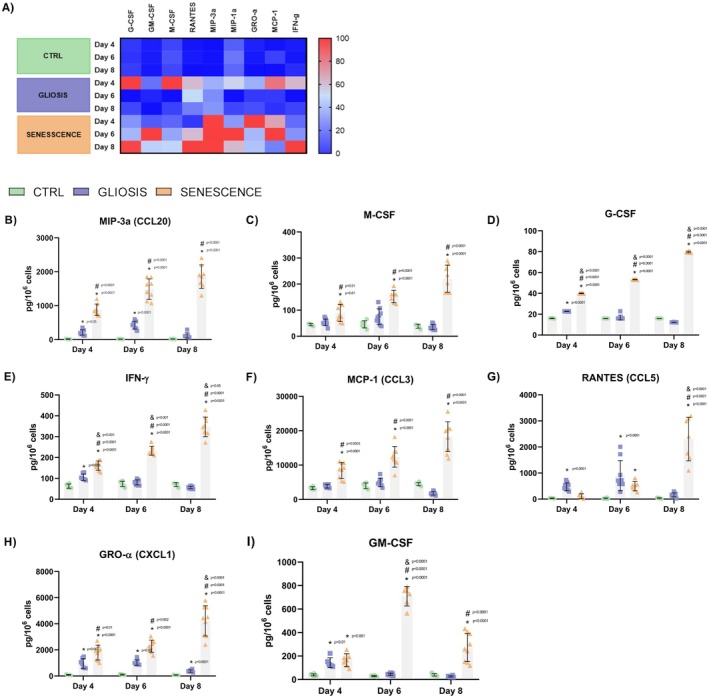
Secretory chemokine profile of reactive and senescent astrocytes on days 4, 6, and 8. Chemokine secretion patterns were analyzed to identify differential inflammatory and chemoattractant signaling between astrocyte phenotypes. (A) Heatmap showing levels of chemokines (G‐CSF, GM‐CSF, M‐CSF, RANTES, MIP‐3α, MIP‐1α, GRO‐α, MCP‐1, IFN‐γ) secreted by control, gliotic, and senescent astrocytes. (B–J) Quantification of individual chemokines revealed phenotype‐specific secretory profiles. Reactive astrocytes showed an early peak on day 4, followed by a decrease. Senescent astrocytes, however, maintained an elevated secretion of chemokines. (ANOVA: *F* = 21.04, *p* < 0.0001, Tukey's: *Q* = 32.39 df = 58, *p* < 0.0001). Data represent *n* = 3 independent biological replicates, each consisting of primary astrocyte cultures derived from pooled cortices of three neonatal rats, with three technical replicates per culture. Concentration values were normalized to cell number to account for differences in cell density across conditions. Significance: *p* < 0.05 vs. control (*), gliotic astrocytes (#), or previous timepoint of same treatment (&).

Chemokine secretion profiles differed (Figure 5A), with reactive astrocytes showing an early peak on day 4, followed by a decrease, especially in MIP‐3α, M‐CSF, and G‐CSF (Figure 5B–D). In contrast, senescent astrocytes progressively secreted higher levels of all chemokines, MIP‐3α, M‐CSF, G‐CSF, IFN‐γ, MCP‐1, RANTES, and GRO‐α (Figure 5E–H) at later time points, except for GM‐CSF (Figure 5I), which peaked at day 6 and then decreased.

Senescent astrocytes showed a higher secretion of cytokines compared to reactive astrocytes when normalized per cell. This model presented a gradual cytokine increase, reaching a peak on day 6. In contrast, reactive astrocytes exhibited a peak on day 4, followed by a subsequent regulation of cytokine levels. Furthermore, senescent astrocytes were characterized by a higher secretion of interleukins within their secretory profile, whereas reactive astrocytes showed a greater release of chemokines.

## 4 Discussion

It is well known that astrocytes can be activated in the face of different stress stimuli and acquire different pathological or physiological phenotypes resulting in a heterogeneity of responses affecting the CNS (Liddelow et al. 2024). The involvement of these different astrocytes´ subsets or phenotypes in the establishment and development of pathology is still not clear, especially in neurodegenerative diseases and during aging. At some point it was attempted to divide reactive astrocytes phenotypes into A1 (associated with damage and neurodegeneration) and A2 (related to the repair response through the secretion of trophic factors), but it is now accepted that the reactive astrocytes heterogeneity is very complex and dynamic. So to determine astrocyte'phenotypes, multiple parameters need to be considered, such as gene expression, morphology, and their function in disease pathogenesis and progression, among others (Escartin et al. 2021; Matusova et al. 2023). Nevertheless, these astrocytes´ phenotypes have not taken into account senescent astrocytes, which also secrete proinflammatory molecules, but, unlike reactive astrocytes, they do not increase their proliferation, but stop it (Maciel‐Barón, Morales‐Rosales, et al. 2018; Maciel‐Barón, Moreno‐Blas, et al. 2018).

Remarkably, Habib et al. (2020), using sNuc‐seq to study the transgenic mice model of AD (5 × FAD), found an additional astrocyte phenotype (besides the regular astrogliosis) characterized by a GFAP‐high content, which they termed disease‐associated astrocytes (DAAs). DAAs were observed in the cortex and hippocampus in both male and female 5 × FAD mice. DAAs were derived from homeostatic GFAP‐low astrocytes and increased with age. Moreover, in WT mice, DAA‐like cells were also found starting at 13–14 months and increased in abundance in 20‐month‐old mice. Interestingly, our results showed that senescent astrocytes have increased levels of GFAP compared to gliosis, concurring with DAA astrocytes and suggesting their resemblance and presence in AD and aging.

There are numerous reports of in vivo and in vitro observations in which reactive astrocytes show intrinsic responses that are difficult to explain (Patani et al. 2023; Ziff et al. 2022; Lee et al. 2023), and that could be understood considering the abundance of senescent astrocytes coexisting with reactive ones. On the other hand, studies of senescence have shown that there are differences in the burden of senescent cells in tissues due to different intrinsic responses of each cell to stress, which would lead to deciding the cell fate: senescence or apoptosis (Yousefzadeh et al. 2020). This would explain the beneficial effects, or lack thereof, of senolytic treatments. Interestingly, these scenarios also do not consider that astrocytes may have another cell fate: astrogliosis. Thus, attempting to eliminate senescent cells to reduce neuroinflammation could promote gliosis, which increases inflammation. Therefore, it is important to understand the involvement of both subsets of astrocytes in the generation of neuroinflammation associated with pathologies and aging in order to address them.

Here we found that, in response to the same stressor (PA), astrocytes entered either senescence or gliosis, each characterized by a distinct set of differentially expressed genes (DEGs) and secretory profiles. While both states shared common pro‐inflammatory features, senescent astrocytes exhibited a greater cytokine production and had genes enriched in inflammatory pathways, suggesting a more pronounced inflammatory state in senescent astrocytes compared to gliotic astrocytes.

### 4.1 Senescent and Gliotic Astrocytes Upregulate Specific Genes and Pathways

In the gliosis model, genes, such as SERPING1, AMIGO2, PSMB28, SRGN, GBP2, and LCN2, were upregulated, all of which have been previously reported in reactive A1 astrocytes by Zamanian et al. (2012), Liddelow et al. (2017), and Hasel et al. (2021), and although that classification has been challenged (Escartin et al. 2021), these markers of inflammation are still commonly used to characterize gliotic astrocytes. Together with the transcripts of pro‐inflammatory cytokines (IL‐11, CXCL3, CXCL12, CXCL6, CX3CL1, MMP2, MMP13) (Simmnacher et al. 2020), which were upregulated. Furthermore, the upregulation of C3, CFB, and GFAP is a significant feature of activated astrocytes (Li et al. 2019), which is consistent with the increase in C3 and GFAP in our transcriptomic results and was supported by the immunofluorescence experiments.

Senescent astrocytes showed characteristic markers, such as IGFBP5, CDKN1A, and p53, which have been previously reported in human senescent astrocytes by Simmnacher et al. (2020) as indicators of cell cycle arrest. These findings align with the proliferation assays, where the senescence in vitro model showed arrested growth. Johmura et al. (2016) demonstrated that FBXO2 is involved in the activation of p53 in senescent cells, while Narita et al. (2006) showed that High‐Mobility Group A (HMGA) proteins accumulate on senescent fibroblast chromatin and are essential structural components of SAHFs. Furthermore, Li et al. (2023) highlighted the role of NF‐kB and CCND1 in SASP secretion, which are crucial for the release of cytokines, such as IL‐6, CXCL1, and IL‐1α, in senescence. All these markers were upregulated in our model.

On the other hand, the classical markers of senescence, such as Lamin B1 and GLB (β‐Galactosidase), were confirmed by immunofluorescence in the senescent astrocytes. Additionally, these genes were corroborated using CellAge: The Database of Cell Senescence Genes, confirming their involvement in various senescence‐inducing stimuli, including stress‐induced, chemotherapy‐induced, replicative senescence, and oncogene‐induced senescence.

A limitation of this study is that some markers are similarly expressed in both gliosis and senescence. Notably, we observed reduced levels of Lamin B1 during gliosis, a protein commonly associated with cellular senescence. Additionally, increased levels of C3, GFAP, and S100a10 were found in senescent astrocytes. These findings warrant further investigation, as they reflect a recurring challenge in the field—particularly regarding senescence—where no single, definitive marker exists. This highlights the need for additional markers, such as those related to cellular proliferation. The absence of proliferation in senescent cells, contrasted with the hyperproliferation seen in gliosis, supports the notion of distinct underlying phenotypes. Further research is necessary to elucidate the rates of proliferation, survival, and cell death in these models. Consequently, future studies should aim to identify specific markers for these biological processes to clarify which events are being affected.

### 4.2 Key Genes and Pathways in Gliotic and Senescent Astrocytes

The transcriptomes of senescent and reactive astrocytes revealed DEGs specific to each phenotype. In gliosis, C3 and LCN2 genes, identified as reactive markers due to their complement activation, doubled in expression compared to senescence. Gliotic astrocytes also upregulated VCAM‐1, which is involved in leukocyte trafficking and neuroinflammation (Sheikh et al. 2023), and FXYD2, the γ subunit of Na+/K+ ATPase, which regulates sodium and potassium transport in human fetal astrocytes (Gegelashvili et al. 2007). Inflammatory markers, such as REG3B, CXCL6, CCL2, CXCL1, and CXCL3, reported in human astrocytes by Simmnacher et al. (2020), were also upregulated. Downregulated DEGs were mainly related to cell cycle dysregulation, contributing to the observed increase in proliferation.

Hence, our transcriptional signature for reactive astrocytes is characterized by the upregulation of inflammatory markers, chemokine production, immune response pathways, complement cascades, and pro‐inflammatory signaling pathways.

In contrast, the top DEGs in the senescent model included FOSB and FOXG1, which are linked to cell cycle regulation and neuroinflammation. FOSB has been implicated in diseases like Parkinson's (Nascimento et al. 2023), while FOXG1 plays a role in aging and inflammation (He et al. 2020). EDNRB (endothelin receptor B) regulates cytoskeleton dynamics, and its reduction has been associated with reactive responses in astrocytes following brain injury (Cheng et al. 2023). The increased expression of EDNRB in senescent astrocytes may indicate the persistent activation of signaling pathways such as JNK, p38MAPK, ERK, and c‐Jun, which are associated with stress responses and cumulative cellular damage. While these pathways are essential in reactive astrocytes to support regeneration after injury, their chronic activation in senescent astrocytes could sustain a pro‐inflammatory environment, ultimately impairing normal repair processes in the central nervous system (Hammond et al. 2015). Moreover, the increased expression of VAT1L in senescent astrocytes may reflect a mechanism similar to those observed in gliomas regarding immune evasion, immunosuppression, and regulation of immune checkpoints (Yang et al. 2021). Although there are no specific studies on VAT1L in astrocytes, its role in immune and inflammatory processes could contribute to the chronic pro‐inflammatory environment characteristic of senescence. Further research is needed to clarify its function in this context.

Other interesting genes, EGR3 and EGR4, both upregulated in response to neuronal activity, are central to schizophrenia risk pathways (Meyers 2020). The genes EGR4, EGR3, EDNRB, VAT1L, and FOXG1 have been reported in glia, but there is no information on them in senescence. The increased expression of EGR3 and EGR4 in senescent astrocytes may be linked to findings from a study describing how EGR3 participates in cell cycle arrest and senescence in response to oncogenic stress, acting through the Arf‐Egr‐C/EBPβ pathway. The study also suggests that EGR3 regulates cell maturation and differentiation, as well as genes related to the extracellular matrix, promoting morphological changes, such as the formation of cellular extensions (Salotti et al. 2015). While EGR4 is less studied, it may have similar functions, contributing to microenvironment remodeling and the pro‐inflammatory state characteristic of astrocyte senescence. Finally, CXCL11, involved in immune‐inflammatory responses and brain injuries (Wu et al. 2018), was also upregulated.

So, the senescent signature included genes that contribute to neuroinflammation, cell cycle arrest, and cytoskeletal reorganization, with pathways activated by NF‐kB and cytokine production.

### 4.3 Cytokine Profiles in Senescent and Gliotic Astrocytes

Several studies have examined the secretion of pro‐inflammatory cytokines in cortical astrocytes treated with PA, but the concentrations are very variable, ranging from very high concentrations, such as 500–250 μM (Ortiz‐Rodriguez et al. 2018), to low concentrations such as 50–75 μM (Fróes et al. 2024). All these studies have reported increased cytokines but have not assessed whether the astrocytes are in senescence or gliosis. So, one of our main objectives was to compare the secretions of senescent and activated astrocytes induced by the same stressor and, thus, confirm the inflammatory results of the transcriptome.

Both senescent and gliotic astrocytes showed increased secretion of IL‐6 and TNF‐α, as previously reported in PA‐treated astrocytes (Hsiao et al. 2014; Vesga‐Jiménez et al. 2022; Gupta et al. 2012; Ortiz‐Rodriguez et al. 2018); but activated astrocytes displayed an increase in IL‐1β secretion on day 2, which Liu and Chan (2014) linked to IPAF inflammasome activation in astrocytes. IFN‐γ increased on day 4; thus, it might have contributed to the reduction of CCL5, IL‐1β, and TNF, as well as GFAP upregulation (Soung and Klein 2020), potentially explaining the decline in IL‐1β and TNF by days 6 and 8. IL‐17α secretion increased on day 4 in both groups, potentially explaining the IL‐6 secretion peak on day 6, as IL‐17α promotes IL‐6 production and potential leukocyte infiltration (Soung and Klein 2020). Other studies in reactive astrocytes have shown that cytokine induction by IL‐1β and TNF‐α primarily increases the secretion of G‐CSF, GM‐CSF, and GROα, followed by MCP‐1. Astrocytes activated by IL‐1β/TNF‐α for 24 h exhibited increased secretion of IL‐1β, TNF‐α, MIP‐1α (CCL3), and RANTES (CCL5) (Choi et al. 2014).

Interestingly, senescent astrocytes also showed anti‐inflammatory activity, with increased IL‐4 and IL‐10 secretion.

Our results show a sustained increase in cytokines and chemokines (IL‐1α, IL‐1β, IL‐6, TNF‐α, CCL2, CCL5, among others) in senescent astrocytes, with peaks around day 6 and levels maintained until day 8. This aligns with previous studies, such as Maciel‐Barón et al. (2016), where IL‐1α stands out as a central component of the SASP, activating other inflammatory pathways. Similarly, chemokines like MCP‐1 and RANTES attract immune cells, suggesting that the SASP in senescent astrocytes aims to recruit immune cells to eliminate damaged cells. Additionally, Maciel‐Barón et al. highlight the dynamic nature of the SASP, which evolves over time, possibly adapting to the microenvironment or the specific state of senescence. This dynamism could explain the changes in cytokine secretion patterns observed in our study.

This dynamic nature of the SASP, observed both in a previous systematic review (López‐Teros et al. 2024) and in our experimental data, is clearly reflected in the secretion patterns of cytokines in senescent and reactive astrocytes. In senescent astrocytes, we observed a sustained increase in molecules, such as IL‐1α, IL‐1β, IL‐6, TNF‐α, CCL2, and CCL5, with peaks around day 6 and levels maintained until day 8. According to the review, these cytokines are key components of the SASP, whose prolonged secretion in senescent astrocytes may be linked to their role in driving chronic inflammation. The review concluded that senescent astrocytes are more often associated with chronic inflammatory processes, while reactive astrocytes primarily play a role in acute inflammatory responses, highlighting a fundamental difference in the dynamics of these two states.

On the other hand, our data showed that reactive astrocytes exhibit an early peak in secretion on day 4 for cytokines, such as IL‐1β, TNF‐α, IL‐6, and IL‐18, along with the same chemokines observed in senescence, such as CCL2 and CCL5, although at significantly lower levels than in senescent astrocytes. This pattern in reactive astrocytes, consistent with the systematic review, reflects their rapid response to acute stimuli. In contrast, senescent astrocytes maintain sustained secretion, contributing to a persistent inflammatory environment. These findings reinforce the idea that reactive and senescent astrocytes contribute differently to the regulation of the central nervous system microenvironment, depending on the duration and intensity of their cytokine and chemokine secretion.

A significant result we found was that the crude levels of cytokines secreted by astrocytes in gliosis were higher than those of senescent astrocytes. However, when normalized for cell number, senescent astrocytes secreted significantly higher concentrations of cytokines. This is very interesting because the brain is likely to contain more reactive astrocytes than senescent, and the contribution of the latter is lower in early life. However, with age or disease, the number of senescent astrocytes would increase, and their contribution could become more important.

### 4.4 Distinct Roles of Reactive and Senescent Astrocytes in Neuroinflammation and Immune Modulation

Astrocytes play a fundamental role in neuroinflammation by secreting signals that enable their direct interaction with innate immune cells, such as microglia, perivascular cells, blood‐derived leukocytes, peripheral‐derived macrophages (PDMs), and neutrophils (Hellenbrand et al. 2021). As mentioned before, this communication is mediated by the release of a wide variety of cytokines, chemokines, and growth factors as part of the innate immune response (Liddelow et al. 2024).

Both astrocytes phenotypes exhibited sustained secretion of chemokines, such as CCL20, CXCL1, CCL3, CCL2, and CCL5, over 8 days, consistent with recent findings from post‐injury rat studies where MIP‐1α, CXCL1, and RANTES were reported to promote the recruitment of monocytes, T cells, and dendritic cells to the injury site (Hellenbrand et al. 2021). Additionally, chemokines, such as CCL2, CXCL1, CCL5, and CXCL10 are known to remodel the CNS microenvironment and to play a crucial role in recruiting T cells, thereby driving local inflammatory responses (Sanmarco et al. 2021).

The importance of CCL2 is particularly noteworthy, as its experimental deletion has been associated with reduced leukocyte infiltration and a shift in macrophage phenotype toward an anti‐inflammatory state, underscoring its key role in regulating inflammation (Linnerbauer et al. 2020). Furthermore, GRO‐α induces the chemotaxis of neutrophils and granulocytes, while RANTES, produced by astrocytes, promotes the migration of M2 macrophages to the injury site, facilitating tissue repair responses (Hellenbrand et al. 2021).

In addition, RNA‐seq results suggest an increase in reactive astrocytes of genes such as CD6, BTNL10, SECTM1B, SH2D1A, IL‐23, STAT4, and CD7, which are associated with the attraction of T lymphocytes. The overexpression of FPR1 was also observed, a gene involved in activating the innate immune response and directing the chemotaxis of neutrophils and monocytes. Moreover, the upregulation of IL‐15 suggests a heightened activity of T cells and NK cells. Interestingly, in senescent astrocytes, a higher expression of genes such as Mpa2l, Prkcb, and Tlr2 was observed. These genes are associated with inflammatory signaling pathways and immune activation, which may indicate a more pronounced role of senescent astrocytes in chronic inflammation and immune regulation. These findings align with the secretory profiles observed; however, further studies are required to confirm these associations and elucidate the underlying mechanisms.

In the context of astrocyte‐microglia interactions, this relationship becomes particularly critical during neuroinflammation. Pro‐inflammatory cytokine secretion by astrocytes, such as IL‐1α, IL‐1β, GM‐CSF, and IL‐6, not only stimulates microglial activation but also enhances microglial proliferation, establishing a bidirectional feedback loop that amplifies the pro‐inflammatory state (López‐Teros et al. 2022; Linnerbauer et al. 2020; Hellenbrand et al. 2021). Additionally, interleukins, such as IL‐1β, IL‐6, and TNF‐α, are directly involved in the recruitment of lymphocytes to the injury site (Linnerbauer et al. 2020).

Astrocytes, in their reactive and senescent states, play different but critical roles in the dynamics of neuroinflammation and immune responses in the CNS. Reactive astrocytes are characterized by higher secretion of chemokines, such as CCL2, CXCL1, CCL5, and CXCL10, which may remodel the CNS microenvironment and promote the recruitment of immune cells, including monocytes, T cells, dendritic cells, and neutrophils, to the site of injury. This state is essential for the initial mobilization of immune cells, driving acute inflammation and facilitating the repair processes. In contrast, senescent astrocytes exhibited higher secretion of pro‐inflammatory cytokines, such as IL‐6, IL‐1α, and TNF‐α, which are associated with microglial activation and lymphocyte recruitment to the site of damage. While reactive astrocytes produce a chemokine‐dominant secretory profile, senescent astrocytes showed a significantly higher secretion of these same molecules in an inter‐model comparison, suggesting a greater impact on chronic inflammation and tissue remodeling.

This comparison highlights that reactive astrocytes are more aligned with acute inflammation and localized signaling, whereas senescent astrocytes contribute to persistent low‐grade inflammation and long‐term modulation of the immune microenvironment. Both states play complementary yet different roles in neuroinflammation and CNS pathophysiology. A question that remains to be answered is the relationship and effect of one phenotype on the other. One hypothesis is that senescent astrocytes may induce senescence in the reactive, thereby promoting neuroinflammation and damage during aging, but this would be the subject of a later study.

The RNA‐seq analysis revealed other notable differences between reactive and senescent astrocytes related to their metabolic and functional roles, such as glutamate homeostasis, lipid and glycolytic metabolism, energy balance, stress adaptation, water homeostasis, and astrocytic plasticity. These findings would be fascinating to explore further in the future.

Finally, understanding that astrocytes can be found in different states is extremely important when administering any pharmacological therapy, especially the use of senolytics during aging, as we do not know what effect they may have on the reactive astrocyte population.

## 5 Conclusion

This study establishes that under the same stressor, but at different concentrations, astrocytes can adopt distinct pathological states—senescence or gliosis—each characterized by its unique transcriptional profile and secretory behavior. While both senescent and gliotic astrocytes share pro‐inflammatory traits, senescent astrocytes exhibit a more pronounced inflammatory phenotype, as indicated by the enrichment of genes and pathways involved in cytokine production and inflammatory signaling, particularly those related to NF‐κB, TNF, and MAPK pathways. Gliotic astrocytes, on the other hand, are more associated with increased cell proliferation, extracellular matrix remodeling, and the activation of complement cascades, which contribute to neuroinflammation and tissue repair processes.

Overall, this study underscores the dual role of astrocytes in driving neuroinflammation, with both senescence and gliosis contributing to the aging process. As the brain ages, these astrocytic states may exacerbate chronic inflammation, or “inflammaging,” which is a key factor in age‐related cognitive decline and neurodegeneration. The heightened secretion of pro‐inflammatory cytokines by senescent astrocytes can contribute to persistent low‐grade inflammation, while the increased chemokine production in gliotic astrocytes may promote immune cell infiltration and tissue damage over time. Understanding the specific pathways and secretory profiles of these astrocytic states during aging offers potential targets for therapeutic interventions to mitigate neuroinflammation and promote healthier brain aging.

## Author Contributions

**Michel López‐Teros:** conceptualization, data curation, investigation, methodology, writing – original draft, writing – review and editing. **Karla Estephanía Ávila‐Galicia:** formal analysis, methodology. **Raúl Librado‐Osorio:** formal analysis, methodology. **Verónica Jimenez‐Jacinto:** data curation, formal analysis, methodology, writing – review and editing. **Jorge Antonio Garcia‐Álvarez:** formal analysis, methodology. **Georgina Hernández‐Montes:** data curation, formal analysis, methodology. **Alejandro Sanchez‐Flores:** data curation, formal analysis, methodology, writing – review and editing. **Adriana Alarcón‐Aguilar:** conceptualization, formal analysis, investigation, methodology, writing – review and editing. **Armando Luna‐López:** conceptualization, data curation, formal analysis, investigation, writing – review and editing. **Mina Königsberg:** conceptualization, funding acquisition, investigation, supervision, writing – original draft, writing – review and editing.

## Ethics Statement

This project was approved by the Universidad Autónoma Metropolitana‐Iztapalapa UAM‐I, ethics committee (dictate 1706‐2018).

## Conflicts of Interest

The authors declare no conflicts of interest.

## Peer Review

The peer review history for this article is available at https://www.webofscience.com/api/gateway/wos/peer‐review/10.1111/jnc.70164.

## Supporting information


Appendix S1.

## Data Availability

The data that support the findings of this study are openly available at: https://drive.google.com/drive/folders/1rHSpFAB9mNWKhbw6hYdqHuox8_8_PzjM and in the Supporting Information.
