# Supplementary material for: Palmitate‐Induced Primary Rat Senescent Astrocytes Exhibit Higher Inflammatory Activity and a Distinct Transcriptomic Profile Compared to Reactive Astrocytes
Source: J Neurochem. 2025 Jul 28;169(7):e70164. doi: 10.1111/jnc.70164 (PMC12302067; doi:10.1111/jnc.70164)
Supplement: Supplementary file 1 — Appendix S1. [file JNC-169-0-s001.zip › jnc70164-sup-0003-Supinfo3.pdf]

PALMITATE-INDUCED PRIMARY RAT SENESCENT ASTROCYTES EXHIBIT HIGHER INFLAMMATORY ACTIVITY AND A DISTINCT TRANSCRIPTOMIC PROFILE COMPARED TO REACTIVE ASTROCYTES.

Running title: senescent and gliotic astrocyte comparison

López-Teros Michel[1,2], Ávila-Galicia Karla Estephanía[1,2], Librado-Osorio Raúl[3], Jimenez-Jacinto Verónica[4], Garcia-Álvarez Jorge Antonio[5], Hernández-Montes Georgina[4,6], Sanchez-Flores Alejandro[4], Alarcón-Aguilar Adriana[2], Luna-López Armando[3], Königsberg Mina[2]

[1]Posgrado en Biología Experimental, División de Ciencias Biológicas y de la Salud. Universidad Autónoma Metropolitana Unidad-Iztapalapa. Ciudad de México. [2]Laboratorio de Bioenergética y Envejecimiento Celular, División de Ciencias Biológicas y de la Salud, Universidad Autónoma Metropolitana Unidad-Iztapalapa, Ciudad de México. [3]Instituto Nacional de Geriatría, San Jerónimo Lídice, Ciudad de México. [4]Unidad Universitaria de Secuenciación Masiva y Bioinformática, Instituto de Biotecnología. Cuernavaca, Morelos. [5] Facultad de Ciencias, Universidad Nacional Autónoma de México. Ciudad de México. [6] Red de Apoyo a la Investigación, Universidad Nacional Autónoma de México (UNAM)-Instituto Nacional de Ciencias Médicas y Nutrición SZ, Ciudad de México.

* Corresponding authors.

* Mina Königsberg, Departamento de Ciencias de la Salud, División de Ciencias Biológicas y de la Salud, Universidad Autónoma Metropolitana-Iztapalapa, C.P 09340, Ciudad de México, México. E-mail address: mkf@xanum.uam.mx

* Armando Luna-López, Depto. Investigación Básica, Instituto Nacional de Geriatría, CP 10200. Ciudad de México, México. E-mail address: allbioexp@yahoo.com

Supplementary Table 1. Quantification of cytokine secretion in control, reactive, and senescent astrocytes on days 4, 6, and 8, expressed in pg/10^6 cells. Measurements are expressed as Mean ± SE. n=3 rep=3.

	CTRL			Reactive Astrocyte			Senescent Astrocyte		
	**Day 4**	**Day 6**	**Day 8**	**Day 4**	**Day 6**	**Day 8**	**Day 4**	**Day 6**	**Day 8**
**CYTOKINE**	**pg/mL protein**	**pg/mL protein**	**pg/mL protein**	**pg/mL protein**	**pg/mL protein**	**pg/mL protein**	**pg/mL protein**	**pg/mL protein**	**pg/mL protein**
**G-CSF**	0.218 ± 0.02	0.200 ± 0.02	0.220 ± 0.03	0.999 ± 1.09	0.119 ± 0.02	0.251 ± 0.10	0.412 ± 0.00	0.477 ± 0.11	0.974 ± 0.79
**GM-CSF**	0.653 ± 0.17	0.627 ± 0.16	0.544 ± 0.04	1.755 ± 2.05	0.728 ± 0.23	0.432 ± 0.35	1.700 ± 0.55	7.220 ± 2.40	3.223 ± 1.44
**M-CSF**	0.780 ± 0.05	0.839 ± 0.16	0.790 ± 0.23	2.336 ± 0.15	0.515 ± 0.18	0.754 ± 0.23	0.925 ± 0.33	1.200 ± 0.43	1.388 ± 0.29
**RANTES**	0.405 ± 0.19	0.549 ± 0.30	0.266 ± 0.09	5.723 ± 3.40	4.633 ± 1.84	2.767 ± 1.23	1.256 ± 0.90	5.690 ± 3.24	9.193 ± 5.16
**MIP-3a**	0.253 ± 0.17	0.274 ± 0.13	0.271 ± 0.10	3.611 ± 1.34	2.927 ± 0.67	3.076 ± 0.93	8.703 ± 1.93	8.748 ± 3.22	8.713 ± 2.90
**MIP-1a**	1.112 ± 0.17	1.301 ± 0.28	1.183 ± 0.04	2.179 ± 1.94	0.436 ± 0.26	0.917 ± 0.35	1.572 ± 1.14	3.697 ± 0.04	2.445 ± 1.04
**GRO-a**	3.430 ± 1.67	2.813 ± 1.01	1.738 ± 0.67	16.02 ± 6.12	5.945 ± 1.81	7.697 ± 1.01	37.8 ± 19.59	16.68 ± 4.50	17.51 ± 6.40
**MCP-1**	25.19 ± 4.66	37.0 ± 17.62	24.41 ± 6.35	56.09 ± 7.28	35.24 ± 9.87	32.77 ± 6.42	87.2 ± 23.63	115.7 ± 43.0	50.9 ± 17.50
**IFN-g**	1.594 ± 0.23	1.524 ± 0.16	1.804 ± 0.27	4.530 ± 2.58	1.396 ± 0.47	2.105 ± 1.09	2.604 ± 1.13	3.049 ± 0.74	6.434 ± 4.17
**VEGF**	1.680 ± 0.65	1.941 ± 0.33	2.570 ± 0.27	2.691 ± 0.95	1.889 ± 0.98	1.998 ± 1.15	3.670 ± 1.81	3.963 ± 1.90	3.588 ± 2.21
**IL-1a**	0.200 ± 0.08	0.172 ± 0.05	0.165 ± 0.03	1.729 ± 0.16	0.165 ± 0.06	0.342 ± 0.13	0.729 ± 0.15	3.015 ± 0.43	1.564 ± 0.22
**IL-1b**	0.903 ± 0.23	0.939 ± 0.15	1.140 ± 0.13	3.384 ± 1.68	0.332 ± 0.09	1.036 ± 0.29	2.577 ± 0.62	1.575 ± 0.39	3.265 ± 0.91
**IL-4**	1.220 ± 0.41	0.635 ± 0.16	0.971 ± 0.04	1.719 ± 0.88	0.657 ± 0.18	1.388 ± 0.59	2.912 ± 1.02	3.484 ± 1.86	1.837 ± 0,68
**IL-18**	7.357 ± 0.25	7.303 ± 0.94	7.210 ± 0.02	11.62 ± 2.46	3.311 ± 0.95	5.892 ± 2.16	14.15 ± 0.15	16.46 ± 6.77	14.24 ± 0.68
**IL-6**	3.325 ± 1.02	3.743 ± 1.35	3.456 ± 1.88	6.516 ± 1.93	12.04 ± 5.88	11.5 ± 10.95	25.71 ± 8.43	40.4 ± 21.72	15.18 ± 6.24
**IL-17a**	1.767 ± 0.09	2.124 ± 0.13	1.848 ± 0.17	2.894 ± 0.51	0.717 ± 0.24	2.092 ± 1.10	4.231 ± 0.70	3.383 ± 1.04	2.605 ± 0.21
**TNF-a**	0.524 ± 0.02	0.489 ± 0.02	0.701 ± 0.01	0.508 ± 0.20	0.275 ± 0.06	0.688 ± 0.24	1.063 ± 0.04	1.356 ± 0.30	1.714 ± 1.29
**IL-10**	1.805 ± 1.20	2.127 ± 1.59	2.212 ± 0.82	1.005 ± 064	0.491 ± 0.09	0.913 ± 0.17	2.302 ± 0.05	2.023 ± 0.62	1.865 ± 0.59
**IL-13**	1.262 ± 0.64	1.269 ± 0.35	1.238 ± 0.30	1.987 ± 1.38	0.831 ± 0.54	0.946 ± 0.66	2.943 ± 1.39	2.391 ± 1.37	1.901 ± 1.06
**IL-12**	2.567 ± 1.38	3.035 ± 1.03	3.588 ± 0.73	2.622 ± 1.32	1.247 ± 0.39	2.249 ± 1.13	4.346 ± 2.26	5.480 ± 1.73	4.173 ± 1.36
**IL-2**	21.33 ± 6.69	19.98 ± 7.68	21.42 ± 5.38	12.08 ± 6.58	4.704 ± 1.05	11.00 ± 1.33	21.32 ± 6,95	31.32 ± 5.61	18.87 ± 8.41
**IL-7**	1.155 ± 0.35	1.206 ± 0.28	1.251 ± 0.19	1.049 ± 0.48	0.404 ± 0.11	0.660 ± 0.19	1.616 ± 0.02	1.438 ± 0.42	1.508 ± 0.30
**IL-5**	3.292 ± 0.84	3.725 ± 0.39	3.427 ± 0.79	3.220 ± 1.47	1.186 ± 0.31	2.870 ± 1.38	5.037 ± 0.01	4.864 ± 1.81	4.747 ± 1.48

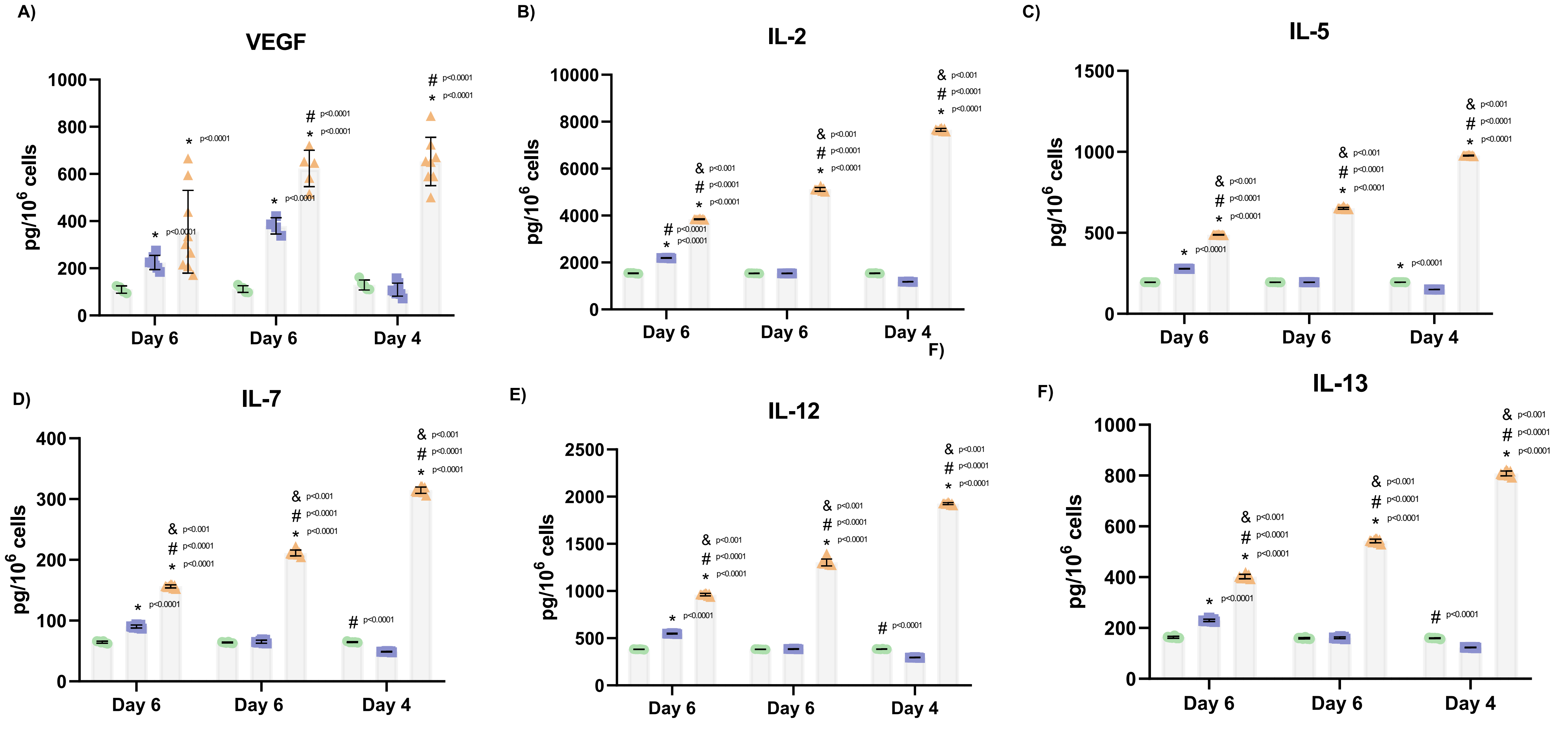

Supplementary Figure 1. Secretory cytokine profile of reactive and senescent astrocytes on days 4, 6, and 8.
Cytokine secretion patterns were analyzed to identify differential inflammatory and chemoattractant signaling between astrocyte phenotypes.
A–F) Quantification of individual cytokines. A) VEGF B) IL-2 C) IL-5 D) IL-7 E) IL-12 F) IL-13. Data represent n = 3 independent biological replicates, each consisting of primary astrocyte cultures derived from pooled cortices of three neonatal rats, with three technical replicates per culture.
Concentration values were normalized to cell number to account for differences in cell density across conditions. One-way ANOVA with Tukey-Kramer post-hoc test was used for statistical comparisons.
Significance: p < 0.05 vs. control (*), gliotic astrocytes (#), or previous timepoint of same treatment (&).
